# Early diagnosis and treatment of breast cancer in Japanese kidney transplant recipients: a single center experience

**DOI:** 10.1186/s40064-015-0946-2

**Published:** 2015-04-25

**Authors:** Taigo Kato, Yoichi Kakuta, Kazuaki Yamanaka, Masayoshi Okumi, Toyofumi Abe, Ryoichi Imamura, Naotsugu Ichimaru, Shiro Takahara, Norio Nonomura

**Affiliations:** Department of Urology, Osaka University Graduate School of Medicine, 2-2 E4 Yamadaoka, Suita, Osaka, 565-0871 Japan; Department of Urology, Tokyo Women’s Medical University Graduate School of Medicine, 8-1 Kawada-cho, Shinjyuku-ku, Tokyo, 162-8666 Japan; Department of Advanced Technology for Transplantation, Osaka University Graduate School of Medicine, 2-2 Yamadaoka, Suita, Osaka, 565-0871 Japan

**Keywords:** Breast cancer, Kidney transplantation, Prognosis, Screening

## Abstract

**Background:**

The incidence of malignancies in kidney transplant recipients is increasing. Breast cancer is a common malignancy after kidney transplantation and can be more aggressive in kidney transplant recipients than in the general population. In this study, we evaluated the incidence and prognosis of breast cancer in kidney transplant recipients.

**Findings:**

Between 1993 and 2013, 750 kidney transplant patients were followed-up at our center. Since 1999, annual physical examination, mammography, and breast ultrasonography have been performed for such patients. Diagnostic studies, including core needle or mammotome biopsy, were performed for suspected malignancies. Patients with malignant neoplasm were administered the appropriate treatment and followed-up to assess tumor response and symptoms.

Nine patients were diagnosed with breast cancer during the follow-up period. The mean age at the initial detection of the breast cancer was 47.7 ± 8.4 years. The mean interval from transplantation to diagnosis was 148.7 ± 37.1 months. Of the 9 patients, 8 were detected through the screening test; 7 were treated with breast conservative surgery and 1 was treated with modified radical mastectomy. The cancer stages were 0 (n = 2), I (n = 6), and II (n = 1). The incidence of breast cancer tended to be unchanged with time between transplantation and diagnosis, inconsistent with the increase in the duration of immunosuppression.

**Conclusion:**

Annual screening tests are crucial in the early diagnosis of breast cancer. Early treatment of breast cancer can result in an excellent prognosis in kidney transplant recipients.

## Introduction

Advances in the development of immunosuppressive agents have significantly reduced the acute rejection rate and markedly improved graft survival in kidney transplantation (Meier-Kriesche et al. 2004). Despite these encouraging trends, the long-term patient survival rate after kidney transplantation has remained unchanged (Webster et al. 2008; Campistol 2009). The high mortality among kidney transplant recipients (KTRs) is attributed mainly to cardiovascular disease and malignancy (Campistol 2009). Over the next decade, the mortality rate from malignancy among transplant recipients will exceed that from cardiovascular disease (Buell et al. 2005). In Europe and North America, the chronic use of immunosuppressants is associated with an increasing rate of malignancy after kidney transplantation (Grulich et al. 2007; Engels et al. 2011; Van Leeuwen et al. 2010). Although the greatest relative increase was observed in the risks of non-melanoma skin cancers and cancers associated with viral infection, the risk of more common solid organ cancers also significantly increased among patients with a relative risk compared with the general population (Webster et al. 2008; Kasiske et al. 2004; Vajdic et al. 2006).

KTRs who develop breast cancer (BC) are often younger at diagnosis and have poorer outcomes than the general population (Buell et al. 2002). BC is the most common cancer and the leading cause of cancer-related death worldwide. Major risk factors include age, family history, and long-term hormonal replacement therapy, which have been strongly linked to cancer stage at diagnosis (Ferlay et al. 2010). In Japanese women between the ages of 40 and 59 years, BC is the leading cause of death, which is considered a growing social problem (Matsuda et al. 2013). However, relatively few studies have addressed the occurrence of BC in KTRs. It remains controversial whether screening tests can reduce BC development after kidney transplantation. In this study, although the number of cases was limited, we aimed to evaluate the efficacy of conducting screening tests for BC in KTRs.

## Patients and methods

### Subjects

Between 1993 and 2013, 750 kidney transplant patients underwent routine follow-up with or without screening at our kidney transplant center. All the clinical data of the KTRs were obtained from our department database. Among the patients, 77 developed de novo malignancy (6 with double cancers) and 9 were diagnosed with BC. Patient characteristics are shown in Table 1. All the patients were closely monitored by performing screening tests. In particular, all the female patients were followed-up annually from 1999 through physical examination, mammography (MMG), and breast ultrasonography (USG). Diagnostic evaluations, including a core needle or mammotome biopsy, were performed for suspected malignancies. Patients with a malignant neoplasm were administered the appropriate treatment and followed-up to assess tumor response and symptoms. All the BC patients were women who underwent surgery between 1998 and 2013.

**Table 1:** **Baseline characteristics of kidney transplant recipients**

	**Total Recipients**	**BC**
Total number	750	9
Age at transplant	38.6 ± 13.3	47.7 ± 2.8
Gender (M/F)	454/296	0/9
Duration to diagnosis (months)		148.7 ± 37.1
Duration of dialysis (years)	3.7 ± 0.2	4.9 ± 0.9
Donor type (Living/Cadaveric)	557/193	7/2
Duration of follow-up (years)	8.8 ± 2.4	6.7 ± 4.9

BC: Breast cancer.

The study protocol was approved by the Institutional Review Board of Osaka University Hospital (approval no. 14150).

### Statistical analysis

Kaplan-Meier estimates were used to calculate patient and graft survival rates. The primary end-point was death (overall survival [OS]). OS was defined as the time from diagnosis of BC to death. Statistical significance was determined using the chi-square test. Differences were considered statistically significant at *p* < 0.05.

## Results

### Malignancy

The overall incidence of malignancy, including multiple primary malignancy, during the follow-up period was 10.3% (77/750 patients). The mean recipient age was 43.6 ± 12.8 years, with 31.4% of the patients aged >50 years. The mean interval from transplantation to diagnosis was 134.5 ± 11.3 months. Other characteristics of the patients are described in Table 1.

### Incidence of BC

BC was diagnosed in 9 female recipients (11.7% of all malignancy cases) but in none of the male recipients. The mean age at the initial detection of BC was 47.7 ± 8.4 years. The mean interval from the time of transplantation to diagnosis was 148.7 ± 37.1 months.

The demographic characteristics of the KTRs with BC are described in Table 2. In 8 of the 9 patients, BC was detected using a screening test. The BC patients underwent breast-conserving surgery (n = 7) or modified radical mastectomy (n = 2). The cancer stages were 0 (n = 2), I (n = 6), and II (n = 1). Four patients received adjuvant endocrine therapy depending on their hormone receptor-positive status. None of the patients received adjuvant chemotherapy. Seven patients received therapy with mycophenolate mofetil and calcineurin inhibitors (CNIs) immediately after kidney transplantation. Of the 9 patients, 2 were administered azathioprine as an antimetabolite. Since 2004, 3 patients have received induction therapy with anti-CD25 antibodies. None of the patients experienced acute rejection episodes. Adjustment of immunosuppressants was not required after BC diagnosis.

**Table 2:** **Characteristics of breast cancers in kidney transplant recipients**

**Patients**	**Age**	**Duration to diagnosis (months)**	**screening test**	**MMG positive**	**USG positve**	**Procedure**	**Pathology**	**Stage**	**Positivity of hormone receptor**	**Recurrence**	**Status**	**Follow-up duration**
1	59	343	+	+	+	CS	solid-tubular carcinoma	1	ER(−),PR(−),HE2(+)	-	Alive	79
2	56	262	+	+	+	CS	solid-tubular carcinoma	1	N/A	-	Dead	56
3	58	270	+	+	+	CS	non invasive ductal carcinoma	0	ER(−),PR(−),HE2(+)	-	Alive	69
4	46	66	-	-	-	MRM	solid-tubular carcinoma	1	ER(−)	-	Alive	187
5	42	57	+	+	+	CS	papillotubular carcinoma	1	HE2(+)	-	Alive	142
6	48	48	+	-	+	CS	papillotubular carcinoma	1	ER(+),PR(−),HE2(−)	-	Alive	113
7	41	113	+	+	+	CS	solid-tubular carcinoma	1	ER(+),PR(−),HE2(+)	-	Alive	24
8	35	80	+	-	+	CS	papillotubular carcinoma	0	ER(+),PR(+),HE2(+)	-	Alive	43
9	44	99	+	+	+	MRM	non invasive ductal carcinoma	2A	ER(+),PR(+),HE2(+)	-	Alive	5

MGM: Mammography, USG: Ultrasonography.

CS: Conservative surgery, MRM: Modified radical mastectomy.

ER: Estrogen receptor, PR: Progesteron receptor, HER2: Human EFGR related-2.

Eight patients were asymptomatic at the time of BC diagnosis, which was detected during the routine screening test. Before the screening system was introduced in 1999, 1 patient felt her own breast mass in 1998. The cancer stages were 0 (n = 2), I (n = 5), and II (n = 1) in the patients diagnosed through the screening test. The patients diagnosed by using the screening test were treated with breast-conservative surgeries (n = 7) or modified radical mastectomy (n = 1).

**Figure 1:**
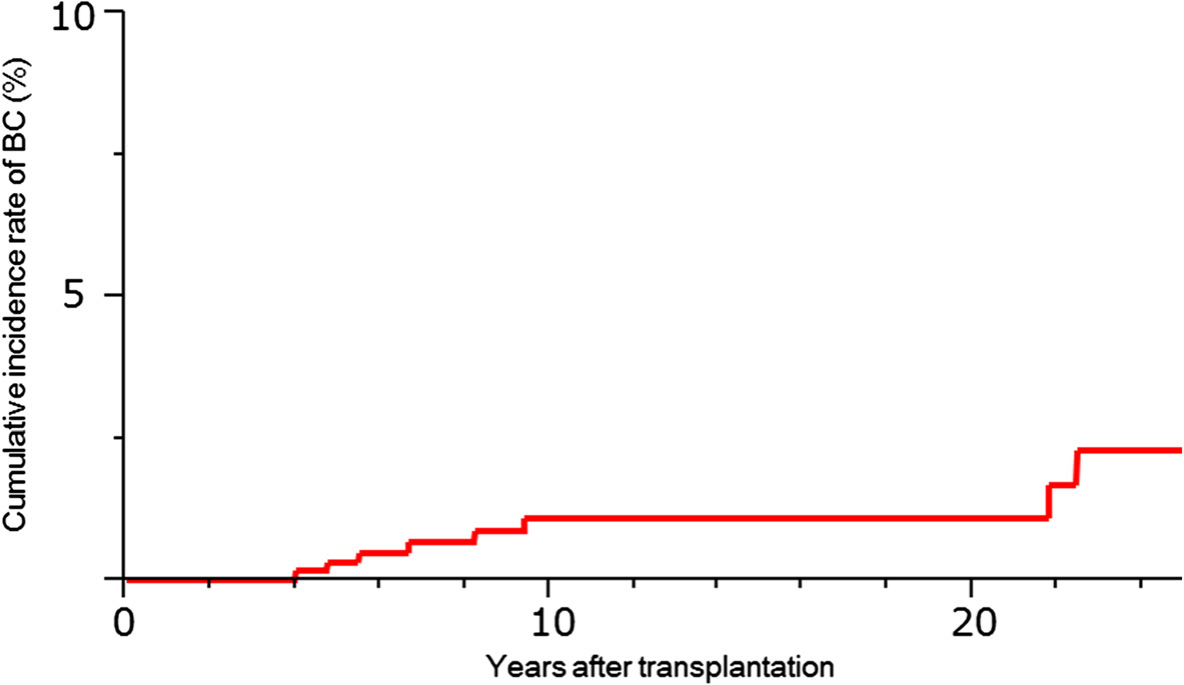
Cumulative incidence rate of breast cancer (BC) after kidney transplantation.

The incidence of BC remained unchanged with time between transplantation and diagnosis, which did not coincide with the increase in the duration of immunosuppressant treatment (Figure 1). After transplantation, BC developed in 2 patients (1.1%) in <5 years, in 4 patients (2.2%) between 5 and 9 years, and in 3 patients (0.8%) at >9 years. The 5-year survival rate was 100% for stage 0, 97.7% for stage I, and 100% for stage II, with no tumor recurrence. After a median follow-up period of 56 months, 1 patient with double cancer died of uterine cancer.

## Discussion

The incidence of cancer after kidney transplantation has increased in recent years due to the increase in patient survival (Meier-Kriesche et al. 2004; Webster et al. 2008; Campistol 2009; Grulich et al. 2007). Cancers in KTRs usually have a more aggressive feature, with rapid progression. The most common cancers in KTRs were non-melanoma skin cancer, urological cancer, and lymphoproliferative disease (Meier-Kriesche et al. 2004; Webster et al. 2008; Campistol 2009; Grulich et al. 2007; Vajdic et al. 2006).

Only few studies have evaluated the incidence of BC post-renal transplantation, although several other cancers have been known to occur more frequently after transplantation (Kasiske et al. 2004; Birkeland et al. 2000; Pedotti et al. 2003; Kauffman et al. 2006). KTRs seemed to have a slightly lower risk of BC than the general population (Agraharkar et al. 2004; Stewart et al. 1995). This could be attributed to the intensive medical screening performed before transplantation, resulting in the exclusion of high-risk patients. Meanwhile, KTRs had undergone long-term dialysis therapy before kidney transplantation. Therefore, given that KTRs require immunosuppressants throughout life, it is necessary to consider the incidence of cancer in dialysis patients as a control group. Vajvic et al. compared the incidence of cancer in KTRs with that in dialysis patients on the waiting list for transplantation. According to the study, kidney transplantation did not increase the risk of other types of cancer including BC although the risk of skin cancer, Kaposi’s sarcoma and malignant lymphoma is increased more than twice in KTRs.

However, Buell et al. also reported that while immunosuppression may not increase the incidence of BC, it might increase the biological aggressiveness of BC (Buell et al. 2002). These reports suggest that early detection of BC may be important for the diagnosis of the disease at an early stage and for initiating the appropriate therapy. In the present study, the incidence of BC among the KTRs was found to be 1.2% at a mean of 148.7 months after transplantation. This rate is higher than that previously reported owing to our strict screening system, which permitted the earlier detection of BC in our study than in previous studies (Buell et al. 2002; Kwak et al. 2013; Popov et al. 2007). It is particularly worth noting that 8 of 9 BC were detected after the screening system was initiated in 1999. Among 8 screening-detected patients, 7 were treated with conservative surgery. One patient who was monitored through screening tests for 8 years underwent bilateral modified radical mastectomy. In this case, the histology was noninvasive ductal carcinoma, which is difficult to detect during screening.

The incidence of BC was unchanged despite the time that lapsed after transplantation in the present study, which is consistent with the previous reports (Buell et al. 2002; Agraharkar et al. 2004; Kwak et al. 2013). This finding suggests that KTRs should continue to undergo long-term screening after transplantation to improve patient outcomes. Generally, the morbidity of BC in Japan is reported to be 103.6 per 100000 which is the most common number in female population and Japanese breast cancer society recommends that women over 40 years should receive BC screening. In the guidelines for the management of transplant recipients, the screening test for BC is recommended for KTRs aged between 50 and 70 years (Kasiske et al. 2000; EBPG Expert Group on Renal Transplantation 2002). However, considering that the mean age at initial detection of BC in our study was 47.7 years, we recommend that patients aged between 40 and 50 years should also undergo the screening test in the same manner as general population.

The benefits of MMG in the detection of BC have long been established (Humphrey et al. 2002). However, MMG has a serious limitation in that its rate of false-negative results can be as high as 35%, especially in women with dense breast tissue (Majid et al. 2003). Moreover, most Japanese women have dense or heterogeneously dense breast on MMG compared to Western women (Takamoto et al. 2013). This can lead to the possibility of missing a cancer lesion. At our center, we also conduct breast USG to compensate for the weakness of MMG and increase the discovery rate of early BC. Interestingly, in some patients, USG clearly showed an irregular and heterogenous nodule though no dominant mass was detected in MMG. In Japan, a large clinical study of adjunct USG is ongoing to address the issue of BC mortality rate reduction (Ishida et al. 2014).

Management of patients by using immunosuppressant drugs after BC diagnosis is a matter of concern. The therapeutic benefit of reducing the dose of immunosuppressant drugs in patients with solid organ tumors is controversial (Self et al. 2006). In some studies, CNI use was not a risk factor of any type of malignancy (Marcen et al. 2003). Moreover, the overall amount of immunosuppressant may not lead to an increased incidence of BC (Buell et al. 2002). These findings suggest that decreasing the dose or discontinuation of immunosuppressants may be unnecessary for the KTRs with BC after diagnosis. In our cases, the dose of immunosuppresants remained unchanged after BC diagnosis.

Renal graft function should be considered before chemotherapy, as some chemotherapeutic agents can affect graft function. Paclitaxel might be an appropriate option for patients with impaired graft function. Data regarding hormonal therapy in these patients are limited.

Recent studies suggest that the use of mammalian targets of rapamycin inhibitors (e.g., sirolimus and everolimus) may reduce the overall risk of solid organ tumors after kidney transplantation (Alberu et al. 2011; Piselli et al. 2013). Furthermore, recent clinical trials reported positive effects of everolimus in improving progression-free survival in women with BC (Baselga et al. 2012). In our center, since 2010, patients with stable renal function who are receiving CNI have been converted to everolimus. The potential protective effect would result in a reduced incidence of BC after kidney transplantation.

Some limitations should be considered when interpreting our results. First, despite our sample size, we had a limited number of BC cases (n = 9) in the KTRs population. Second, the median follow-up period for the KTRs with BC was inadequate. Finally, this was a retrospective study; therefore, we could not control for all confounding variables. However, it is notable that this is the first study to evaluate the incidence of BC in Japanese KTRs.

In summary, given an appropriate screening program, KTRs with BC can be successfully treated using a minimally invasive modality. Although our sample size was small, our experience shows that the patient prognosis in BC is excellent if treatment is administered during the initial stages. We highly recommend BC screening for KTRs to facilitate early detection of BC.

## Consent

Written informed consent was obtained from the patient for the publication of this report and any accompanying images.
